# Ectocytosis renders T cell receptor signaling self-limiting at the immune synapse

**DOI:** 10.1126/science.abp8933

**Published:** 2023-05-25

**Authors:** Jane C. Stinchcombe, Yukako Asano, Christopher J.G. Kaufman, Kristin Bohlig, Christopher J. Peddie, Lucy M. Collinson, André Nadler, Gillian M. Griffiths

**Affiliations:** 1 Cambridge Institute for Medical Research, Keith Peters Building, Cambridge CB2 0XY; 2 Max Planck Institute of Molecular Cell Biology and Genetics, 01307 Dresden, Germany; 3 The Francis Crick Institute, 1 Midland Road, London NW1 1AT

## Abstract

Cytotoxic T lymphocytes (CTLs) kill virus-infected and cancer cells via T cell receptor (TCR) recognition. How CTLs terminate signaling and disengage to allow serial killing has remained a mystery. TCR activation triggers membrane specialization within the immune synapse including the production of diacylglycerol (DAG), a lipid that can induce negative membrane curvature. We found that activated TCRs were shed into DAG-enriched ectosomes at the immune synapse rather than internalized via endocytosis, suggesting that DAG may contribute to the outward budding required for ectocytosis. Budding ectosomes were endocytosed directly by target cells, thereby terminating TCR signaling and simultaneously disengaging the CTL from the target cell to allow serial killing. Thus, ectocytosis renders TCR signaling self-limiting.

The ability to kill multiple target cells in rapid succession is an important factor in the effectiveness of cytotoxic T lymphocytes (CTLs), both in the immune response and in cancer immunotherapies. Such serial killing requires a carefully controlled transition from recognition to detachment from each target cell encountered. The TCR-CD3 complex is formed by T cell receptors (α/β) that recognize peptide-Major Histocompatability Complex I (pMHCI), and a cluster of CD3 chains (γ/δ/ε/ζ) required for signaling (fig. S1A). TCR engagement with pMHCI on target cells triggers rapid phosphorylation of the TCR complex via kinases Lck and Zap70, which initiates downstream signaling (reviewed in (1)). TCR activation can downregulate surface TCR expression via endocytosis (reviewed in (2)) and studies using artificial immunological synapses on lipid bilayers have also shown that TCR can be shed in exosomes (3), and right-side-out microvesicles (ectosomes) (4, 5). What determines the route of TCR downregulation is not known.

## Tracking the T cell receptor in the immune synapse

**Fig. 1:**
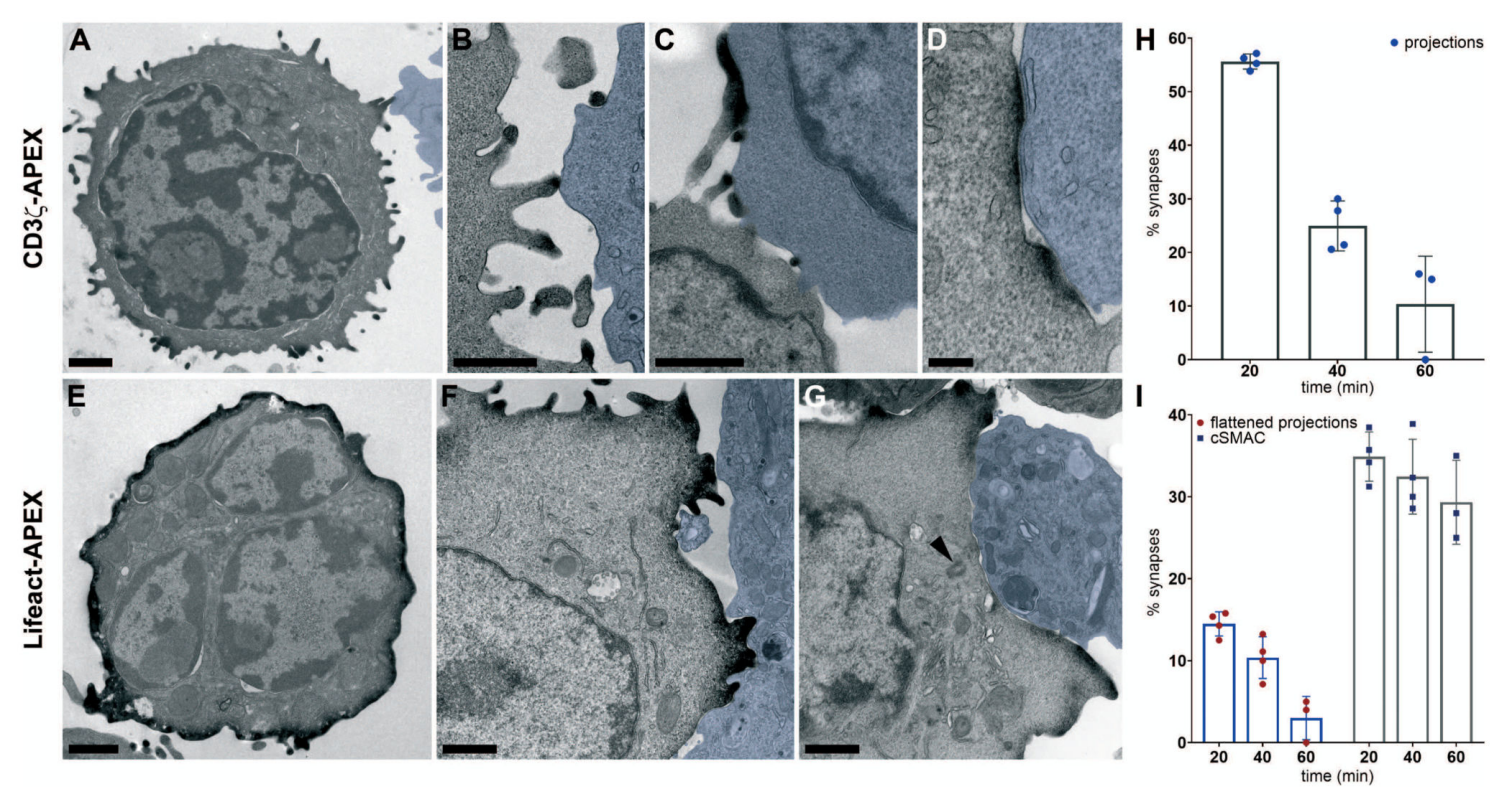
CD3ζ-APEX and Lifeact-APEX localization in CTLs Transmission EM (TEM) images of (**A** to **D**) CTLs expressing CD3ζ-APEX incubated with target cells (blue) for 20 min showing (**A** and **B**) projections, **(C)** flattened projections and **(D)** cSMAC. (**E-G**) CTL expressing Lifeact-APEX with cells in (**F** and **G**) incubated with target cells (blue) for (F) 40 min and (G) 20 min; arrowhead marks centrosome. All 50-70 nm sections, except **(C)** 250 nm. Scale bars, 1 μm (D, 200 nm). Representative of (A) 38, (B) 42 **(C)** 54, **(D)** 67 examples from 4x or (E) 54, (F) 95, (G) 28 examples from 5 independent experiments. (H and I) Percentage of immune synapse images from CTLs and targets incubated for 20, 40 and 60 minutes showing CD3ζ-APEX in (H) projections and (I) flattened projections (red circles) or cSMAC (blue squares) from 107 (20min), 146 (40min) and 49 (60min) APEX-labeled immune synapses. Error bars show mean values ± SD from 4 independent experiments.

To understand how the seamless transitions from TCR recognition to signaling and detachment might be linked, we tracked the fate of TCRs at different stages of immune synapse formation using high-resolution 3D imaging. We labeled CD3ζ with engineered pea ascorbate peroxidase (APEX)(6) (fig. S1A) and imaged TCR localization using electron microscope (EM) tomography. CD3ζ-APEX co-localized with endogenous CD3ε in CTLs (fig. S1B). CTLs engaged targets at different times after mixing, allowing us to capture different stages of immune synapse formation up to the point of fixation in our samples. CD3ζ-APEX, identified by its electron-dense reaction product, labeled the cell surface including the tips of lamellipodial projections (fig. S2, A and B) that make first contact with target cells (Fig. 1 A and B) (7). Our data do not exclude CD3ζ also being present elsewhere on the CTL membrane. These projections flattened against the target (Fig. 1C) and were lost over time as the two cells formed extended areas of close membrane contact, lined by CD3ζ-APEX (Fig. 1D, H, I).

LifeAct-APEX that binds to F-actin was associated with the cortex of isolated CTLs (Fig. 1E) and in lamellipodial projections contacting targets (Fig. 1F) but was depleted where the immune synapse formed an area of tight membrane contact opposite the site of centrosome polarization (Fig. 1G). This was also seen in 3D using focused ion beam scanning electron microscopy (FIB-SEM) (fig. S2 C to E and movie S1). Thus, TCRs in actin-rich lamellipodia make the first contact between CTLs and targets, rapidly flattening to form close membrane contacts lined with TCR, and depleted of actin as the synapse forms.

## Ectocytosis rather than endocytosis at the immune synapse

**Fig. 2:**
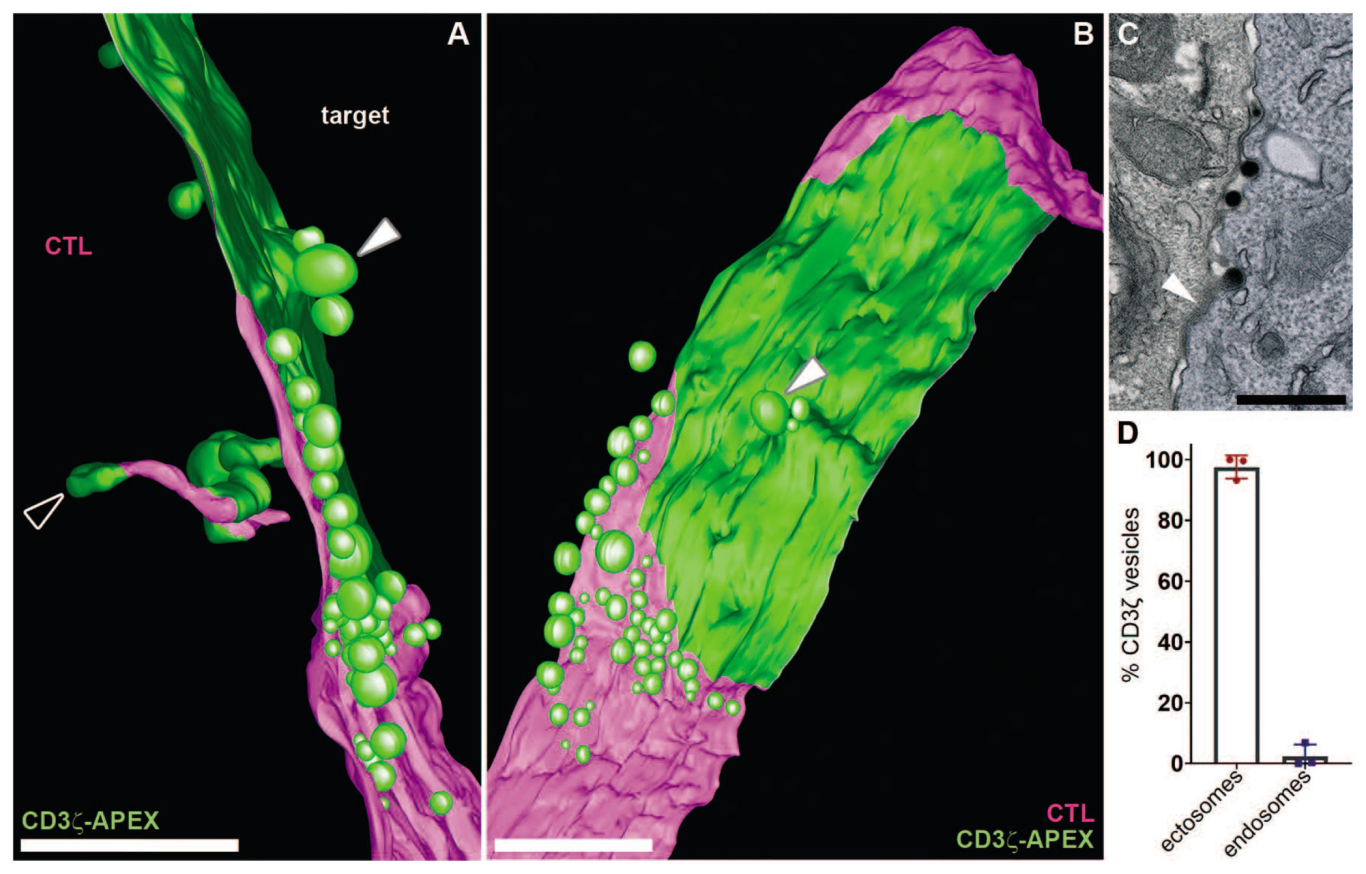
TCR is downregulated via ectocytosis rather than endocytosis across the synapse (**A** and **B**) Images from the 3D model generated from a tomogram reconstruction through ~1 μm across the immune synapse (movie S2) and associated 3D model (movie S3), showing the CTL membrane (magenta) and CD3ζ-APEX labeling (green) with (A) side and (B) en face views. White arrowheads identify the same ectosome in the two different views; white-edged arrowhead a tubular endosome. Representative of 6 independent tomograms. **(C)** TEM image of CTL-target conjugate showing CD3ζ-APEX in protrusions and buds emerging from the edges of membrane clustered CD3ζ (white arrowhead) after incubation with target cells (blue) for 40 min. Representative of 61 examples from 5 independent experiments. Scale bars, 500 nm. **(D)** Percentage of CD3ζ-APEX-labeled endosomes versus ectosomes at the immune synapse. Error bars show mean values ± SD from 3 independent experiments.

We examined the 3D localization of CD3ζ-APEX in 12 separate tomograms each spanning 1 μm across the immune synapse using high-resolution EM tomography (Fig. 2, A to B and movie S2). Modelling of the reconstructed tomograms (movie S3) showed CD3ζ-APEX (green) extending across an area of up to 2 μm in length and >1 μm width on the CTL membrane (magenta) corresponding to TCR clusters seen by light microcopy termed ‘central supramolecular activation clusters (cSMACs)’ (8) where TCR and pMHCI are engaged and CTL and target membranes are tightly apposed. Although we could find CD3ζ-APEX associated with clathrin coated invaginations and tubules internalizing into the CTL, the majority of CD3ζ-APEX was present in 37-210 nm diameter ectosomes (9), budding outward from the edges of the CD3ζ-APEX-labeled cSMAC (Fig. 2, A to C and movies S2 and S3). Quantitation revealed 98% of CD3ζ in ectosomes (Fig. 2D), suggesting that TCR is downregulated via ectocytosis rather than endocytosis across the immune synapse.

## Functions of ectocytosis

**Fig. 3:**
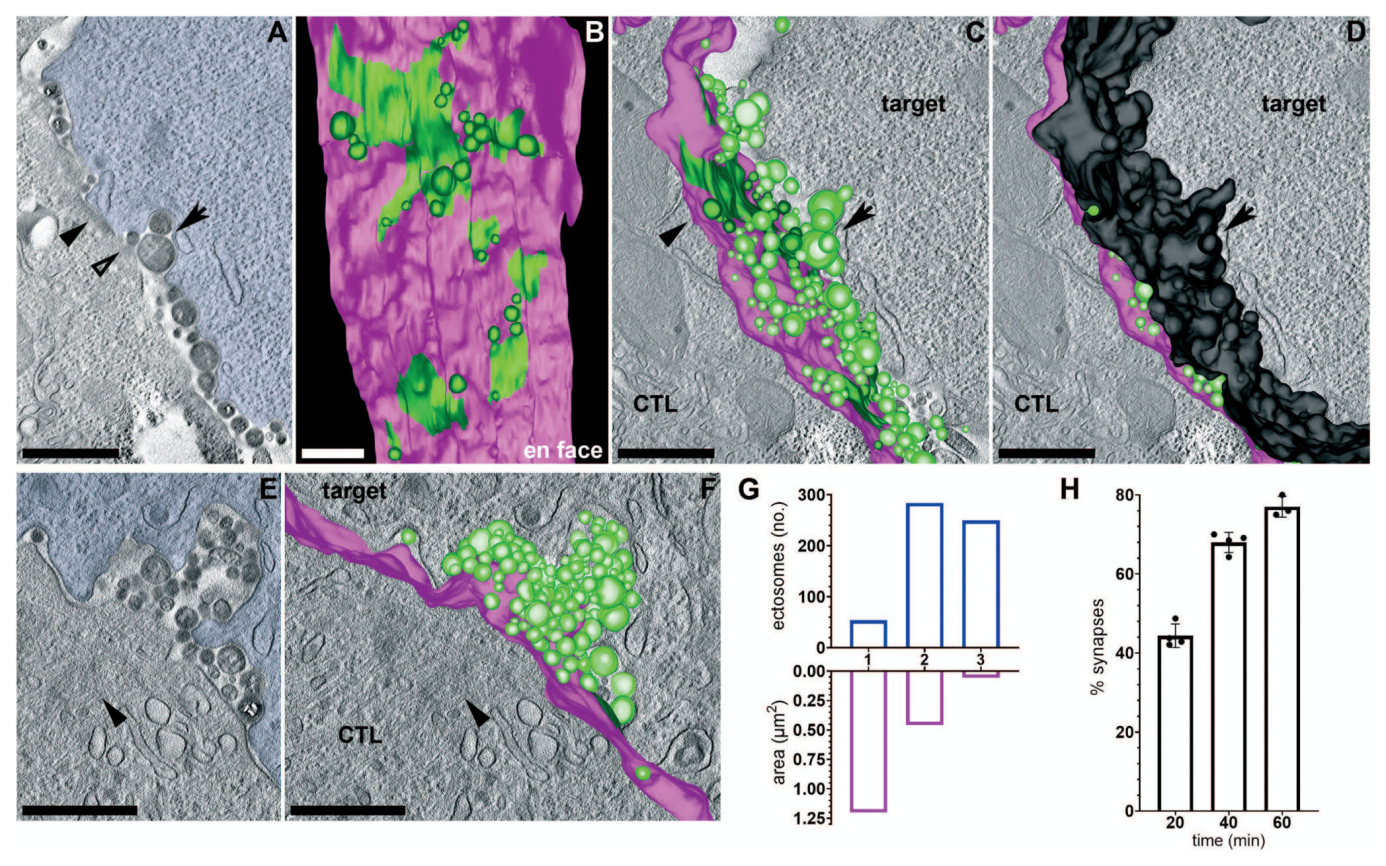
Supramolecular activation clusters (cSMACs) diminish via ectocytosis (**A** to **D**) Images from a tomogram reconstruction through ~1.25 μm across the immune synapse (movie S4) and associated 3D model (movie S5) with CTL membrane (magenta), CD3ζ-APEX (green) and targets false-colored blue or target membrane modelled in black. (A) Single plane from the tomogram series. (B) En face 3D model view showing CD3ζ-APEX plasma membrane labeling plus ectosomes budding from the CTL surface. (C and D) Single tomogram planes with the 3D model superimposed, **(D)** including target membrane. Black arrowheads point to the same area of CD3ζ-APEX-labeled CTL membrane in (A) and **(C)**, and arrows to the same target cell invagination in (A), **(C)** and **(D)**. Open arrowhead shows a budding ectosome in (A). (E and F) Single images from a tomogram series through ~1 μm across the immune synapse (movie S6) with (E) the target cell false-coloured blue and (F) the 3D model (movie S7) superimposed showing the CTL membrane (magenta) and CD3ζ-APEX labeling (green). Black arrowheads indicate the polarized centrosome. Images are representative of 6 independent tomograms. Scale bars, 500 nm. (G) Comparison of total surface area of membrane labeled with CD3ζ-APEX (magenta) with the number of CD3ζ-APEX-labeled ectosomes (blue) across each tomogram. Data for (1) from movie S3 (2) movie S5 and (3) movie S7. (H) Percentage of immune synapses (synapses) with CD3ζ- APEX within ectosomes at different times after cell mixing. Data as described in Fig. 1H and I from 107 (20min), 146 (40min) and 49 (60min) APEX-labeled immune synapses. Error bars show mean values ± SD from 4 independent experiments.

**Fig. 4:**
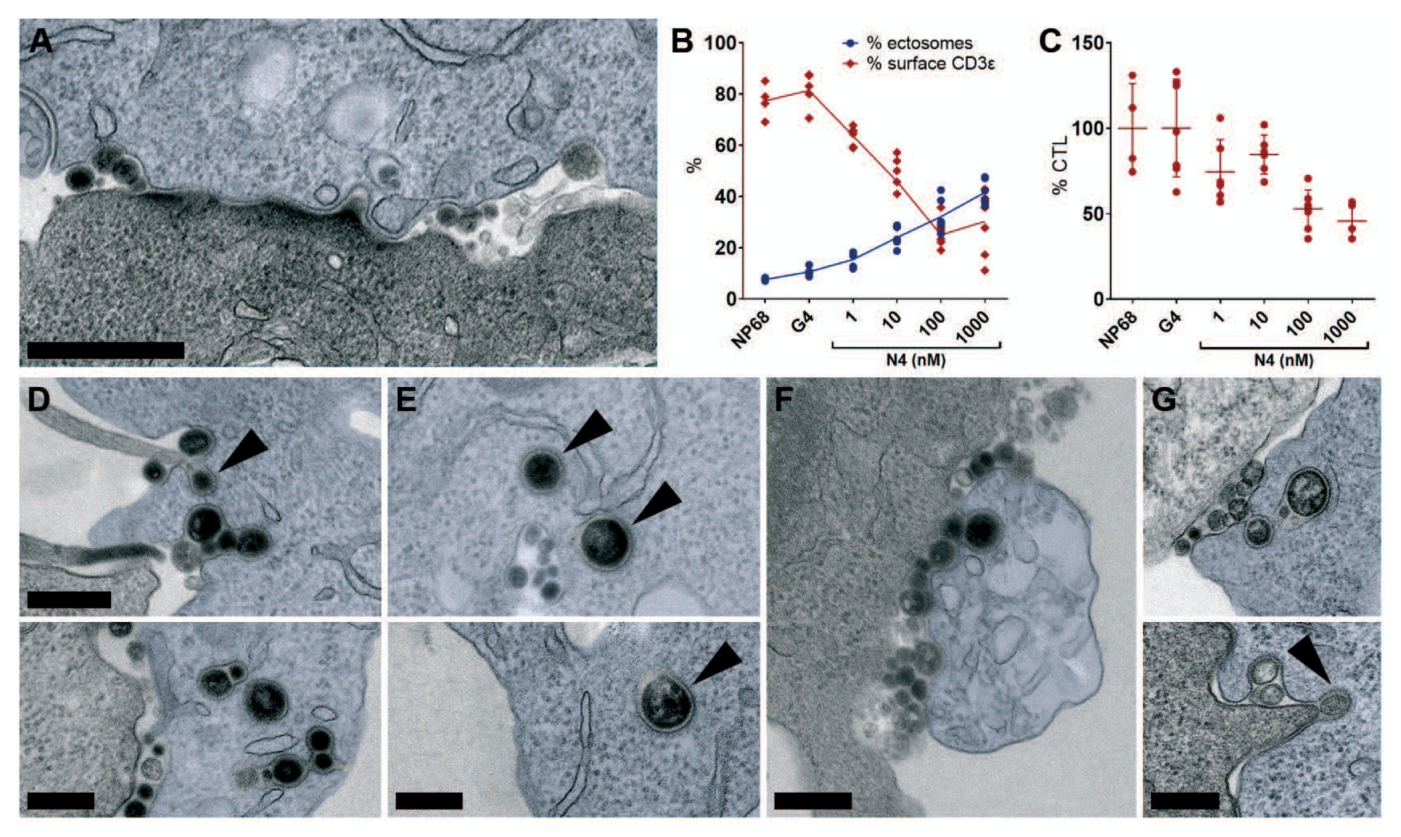
Ectosomes are internalized into target cells by clathrin-mediated endocytosis (A) TEM image through an immune synapse with target cell in blue, showing CD3ζ-APEX ectosomes budding at the edges of membrane-clustered CD3ζ (cSMAC) causing separation of CTL and target membranes. Representative of 61 examples from 5 independent experiments. Scale bar, 500 nm. (B) Percentage CTL with CD3ε surface labeling analyzed as shown in (fig S3) (red graph), and percentage target cells decorated with transferred CD3ε-labeled ectosomes (blue graph), from CTL-target conjugates stimulated with irrelevant (NP68), weak (G4) or strong (N4) peptides. Each data point shows quantitation per 40x field from 163-358 CTL and 274-463 targets per condition, error bars show mean value ± SD from 4-5 technical replicates; representative of 3 independent experiments. **(C)** CTL detachment from CTL-target conjugates over 35 minutes, expressed as percentage of CTLs in NP68 sample (n = 51/field). Data sets as in B. (D to F) TEM images of CD3ζ-APEX expressing CTL showing CD3ζ-ectosomes internalized into target cells (blue) including (F) dying cells with vacuolated ER. Black arrowheads indicate clathrin coats. (G) TEM images of immune synapses between untransfected CTL and targets (blue) showing ectosomes internalized into endocytic vesicles with clathrin coats (black arrowhead). All images are from 50-70 nm sections. Representative of (D to E) 91, (F) 28 synapses from 4 independent experiments, and (G) > 50 synapses from 7 independent experiments. All CTL-target conjugates were fixed after 40 minutes or (F) 60 minutes co-incubation. Scale bars, 250 nm.

3D views and models of tomograms with immune synapses enriched in CD3ζ-ectosomes (Fig. 3, A to F and movies S4 to S7), revealed multiple smaller regions of membrane-associated CD3ζ (42 x 250 – 800 x 250 nm) with ectosomes either budding from the periphery of cSMACs (Fig. 3B) or detached from the CTL membrane (Fig 3. A and C and E and F). Quantitation demonstrated that membrane-associated CD3ζ decreased as ectosomes increased (Fig. 3G) and the number of conjugates with CD3ζ-ectosomes increased over time (Fig. 3H), suggesting that the reduction in area of TCR across the membrane was the result of ectocytosis. Both 3D tomograms and conventional EM also revealed that the tight membrane contacts between CTL and targets were lost where ectosomes budded from the CTL, creating localized cell separation between CTL and target (Fig. 3 and 4A). Quantitation using light microscopy showed ectocytosis increased with TCR signal strength while CTL surface TCR expression decreased (Fig. 4B and fig. S3). TCR signal strength also increased detachment of CTLs from targets (Fig. 4C). Together these results suggest that ectocytosis both removed TCR from the immune synapse and initiated detachment between CTL and targets as TCR ectosomes were shed.

## Fate of shed ectosomes

Our EM tomograms also revealed the fate of shed ectosomes. We found that CD3ζ-ectosomes were closely associated with the target cell membrane, with 83% (n=29) of budding and 71% (n=293) of budded ectosomes in contact with the target membrane as ectosomes separated from CTL (Fig. 3 and 4A, and movie S5). Using CTL-target conjugates we found that ectosomes were taken up into target cells with invaginations containing CD3ζ-ectosomes reaching deep into the target cell (Fig. 4, D to F and fig. S5). These were often decorated with clathrin coats, sometimes with endoplasmic reticulum closely associated (Fig. 4E). CD3ζ-ectosomes were also seen in vacuolated targets that appeared to be dying (Fig. 4F). Similar results were observed with untransfected CTL (Fig. 4G). Thus, budding ectosomes were taken up into dying targets in clathrin-decorated structures.

**Fig. 5:**
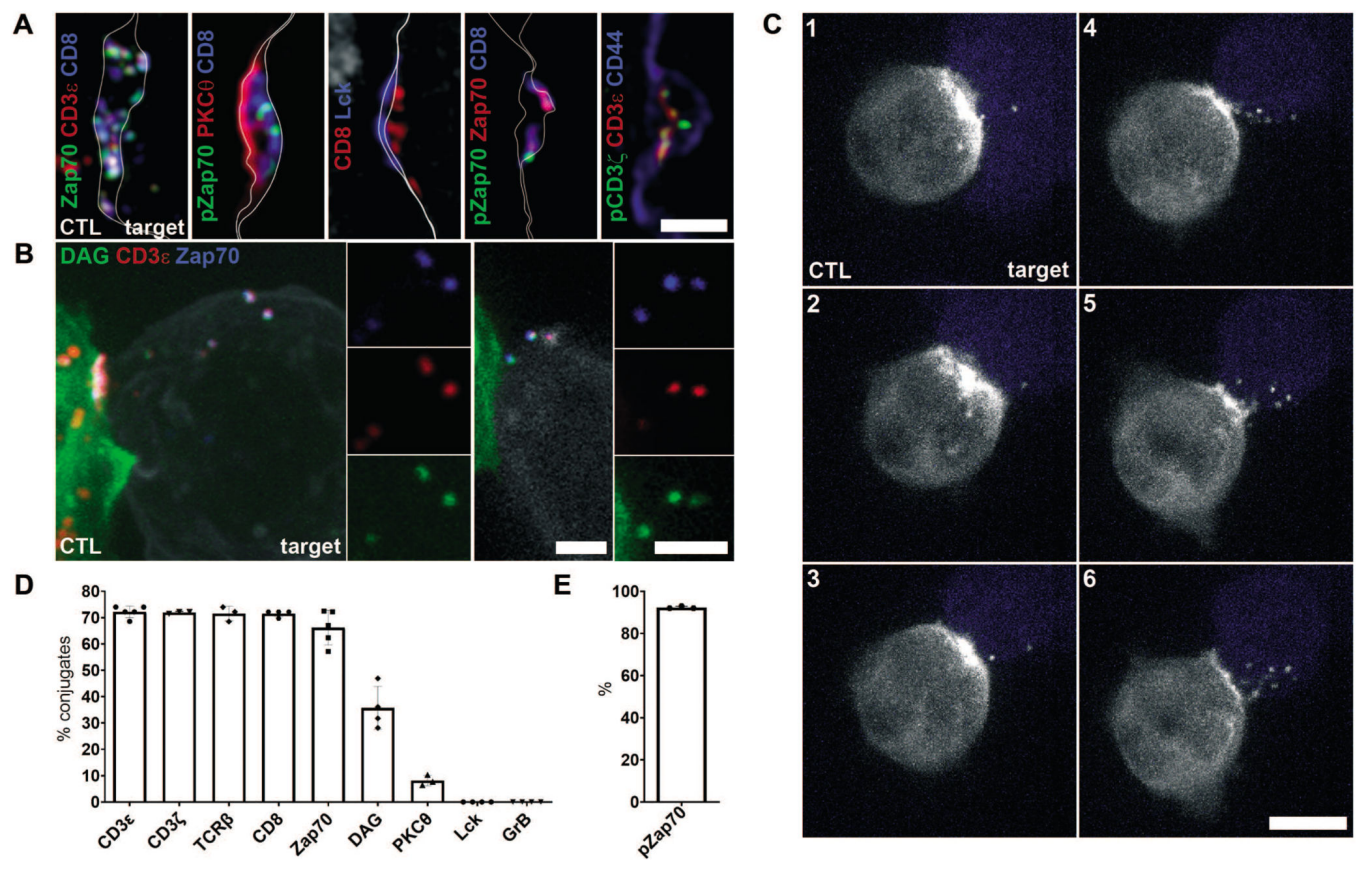
Activated TCR and Zap70 are shed into ectosomes **(A)** SIM imaging showing the central area of the immune synapse between untransfected CTLs and target cells labeled with antibodies as indicated. All panels show a single section image with CTL (left) and target (right) membranes either traced in white or delineated by CD44 labeling of both CTL and target (blue). Images representative of (left to right) 72, 17, 98, 45, 25 synapses from 3-5 independent experiments. Scale bar, 1 μm. (**B**) Projected z-stack (left) and single (right) confocal images through synapses between CTL expressing EGFP-tagged bio-probes for DAG (PKCε, left; PKCγ-C1, right; green) and targets (white), and labeled for CD3ε (red) and Zap70 (blue). Images are representative of 29 (PKCε) and 27 (PKCg-C1) synapses across 2 and 4 independent experiments, respectively. Scale bars, 2 μm. (**C**) Image series from live imaging of CTLs expressing PKCε-EGFP bio-probe for DAG (white) synapsed with target cells (blue) (movie S8). All images are projections from 33 z-planes, 68 sec apart. Data is representative of 6 movies from 4 independent experiments. Scale bar, 5 μm. (**D**) Percentage ectosomes transferred to target cells after 35-45 minutes incubation with CTLs, analyzed by confocal microscopy with each marker as shown. Note, EGFP-tagged DAG bio-probe signal will diminish in acidic endocytic compartments. (**E**) Percentage of Zap70 or CD8 ectosomes also labelled with pZap70. Each data point represents a separate biological repeat (3-5 per marker) and includes data from > 1034 (CD3ε), 158 (CD3ζ), 152 (TCRβ), 180 (CD8), 492 (Zap70), 144 (DAG), 374 (PKCθ), 289 (Lck) and 114 (GrB) and 57 (pZap70) synapses labeled as indicated. Additional representative confocal images used in quantitation are provided in figures S3, S5 and S6.. Error bars show mean values ± SD from 3-5 independent experiments.

To understand whether ectocytosis effectively downregulated signaling from receptors that were shed we asked whether other proteins associated with TCR activation were contained in ectosomes. Using confocal and structured illumination microscopy (SIM) we imaged ectosomes shed from untransfected CTLs. Endogenous CD3ζ, CD3ε, TCRβ, CD8 and Zap70 were all detected in ectosomes that transferred to target cells across the synapse (Fig. 5, A and D and fig. S4 and S5 and S6). By contrast, Lck, the kinase that initiates TCR activation remained clustered on the CTL membrane at the synapse (Fig. 5, A and D and fig. S6). We next asked whether activated TCRs were shed into ectosomes, using antibodies against their phosphorylated forms. Activated pCD3ζ and pZap70 were localized in ectosomes with no evidence of Zap70 in intracellular vesicles within the CTL (Fig. 5A and fig. S7). Thus, released ectosomes contained activated TCRs and Zap70 but not the initial activating kinase Lck, preventing further activation and supporting the idea that ectocytosis can act as a mechanism to terminate signaling from activated TCRs that are shed.

We asked whether ectocytosis of TCRs was linked to the exocytosis of cytolytic granules that is triggered by TCR activation. Rab27a/b-deficient CTLs are unable to release their cytolytic granules, fail to kill target cells and continue to recognize targets over prolonged periods (10, 11). Nevertheless, we found that conjugates formed by Rab27a/b-deficient CTLs generated ectosomes that were transferred across the synapse to target cells (fig. S8). We also confirmed that the cytolytic granule protein granzyme B did not co-localize with CD3ε within CTL and was not found in ectosomes (Fig. 5D and fig. S4). Thus, ectocytosis is independent of cytolytic granule ectocytosis and ectosomes do not contain cytolytic proteins.

## The role of DAG in ectocytosis

We have previously shown that TCR activation creates an area of membrane lipid and phosphoinositide specialization within the immune synapse upon PLCγ cleavage of PIP2, generating DAG (12) (fig. S1A). Intriguingly studies on neutrophils, in which the term “ectocytosis” was first used to define the generation of right-side-out plasma membrane vesicles, demonstrated the preferential sorting of both DAG and specific proteins into shed ectosomes (9). Furthermore, in erythrocytes accumulation of DAG was proposed to create negative membrane curvature, forcing outward budding of vesicles from the plasma membrane (13, 14). We therefore asked whether DAG might contribute to ectocytosis of TCRs from CTLs. To visualize DAG in both fixed and live CTL-target conjugates we used PKC-γ or ε bio-probes that bind DAG (12, 15, 16) (fig. S9 and movie S8). We found that DAG appeared across the immune synapse and separated into ectosomes that co-labeled with anti-TCR antibodies (Fig. 5B). Using live-cell imaging we were able to image DAG-ectosomes emerging from the synapse as DAG was generated during target cell recognition (Fig. 5C and movie S9). Thus, DAG is a membrane constituent of TCR-ectosomes and may contribute to the negative membrane curvature required to initiate ectocytosis of TCRs from CTLs.

Together our findings support a model in which the negative membrane curvature induced by the generation of DAG during TCR signaling could initiate ectocytosis of activated TCRs (fig. S10). We propose that the production of DAG around activated TCRs would contribute to the negative membrane curvature required to initiate ectocytosis, linking TCR activation to the shedding of activated TCRs. Ectosome budding also initiates localized detachment between CTL and target within the immune synapse allowing CTLs to detach from targets once no additional TCRs are recruited and target recognition has ceased.

This model of DAG-driven ectocytosis does not preclude a potential role for Vps4 in ectosome scission (5). However, our experiments showed that expression of dominant negative Vps4 caused TCR to be retained within enlarged endosomal compartments in the CTL thereby preventing synapse formation and Lck recruitment (fig. S11). Consequently, a potential role for Vps4 in ectocytosis cannot be resolved. However, our findings are consistent with potential roles for actin in cell separation (17, 18), as actin is depleted and restored in tune with TCR activation (12).

## Discussion

Using EM tomography we generated 3D views over 1 μm depths across the immune synapse formed between CTLs and targets. Together with >800 TEM and >8000 immunofluorescence images we examined the fate of the TCR within the immune synapse. We found that activated TCRs were shed into DAG-enriched ectosomes at the immune synapse rather than internalized via endocytosis. Ectosomes remained tightly bound by target cells during budding and were internalized into clathrin-decorated structures within the targets. As ectosomes budded from the CTL they created an area of cell separation allowing CTLs to detach from their targets as TCR signaling ceases. Both ectocytosis and CTL detachment increased with signal strength.

Our results provide insights into the well-established phenomenon of TCR downregulation after activation (19–22). Although TCR endocytosis and subsequent degradation was considered the key mechanism for TCR downregulation (2, 19–23), we now show that this is not so at the immune synapse where activated TCRs are preferentially shed via ectocytosis into DAG-enriched ectosomes. Two lines of evidence support our observation that endocytosis of activated TCR is inhibited across the synapse. Firstly, structural studies have demonstrated that adapter protein-2 (AP2) needs PIP2 in the membrane to recruit clathrin and initiate endocytosis (24). Thus, after TCR activation when PIP2 is depleted across the immune synapse (12), AP2 will be unable to initate endocytosis. Furthermore, it has been shown that AP2 cannot bind YXXΦ endocytosis motifs when the tyrosine is phosphorylated (25, 26). Because the 20 functional YXXΦ endocytosis motifs are embedded in immunoreceptor tyrosine-based activation motifs (ITAMs) in which the tyrosine residues are phosphorylated during TCR activation (1, 27), activated TCRs will not be recognised by AP2. Thus, at the CTL synapse endocytosis will be inhibited while ectocytosis will be favored following activation.

Collectively our findings point to two important roles for ectocytosis. Firstly, ectocytosis removes activated TCRs from the membrane of CTLs, terminating signaling as ectosomes are taken up by dying targets. Secondly we find that TCR ectocytosis allows CTL and target to disengage with areas of separation arising naturally as ectosomes bud off from the CTL membrane (see Figs. 3A and 4A). While these might only be small areas of separation during ongoing TCR recognition of the target, as target recognition ceases ectocytosis would allow complete separation and detachment, facilitating serial killing.

Taken together, our findings show that activation induced membrane specialization switches biological function within the immune synapse. These findings suggest that TCR signaling is self-limiting via a process that seamlessly links receptor signaling with shedding of activated TCRs and CTL detachment. We propose that this may provide a general mechanism for shedding receptors post-activation that is likely to be used in many biological systems including cilia (28–32).

## Supplementary Material

movie S1

movie S2

movie S3

movie S4

movie S5

movie S6

movie S7

movie S8

movie S9

Supplementary Materials

## Data Availability

all data are available in the manuscript or supplementary materials.

## One-Sentence Summary

Activated T-cell receptors are shed via ectocytosis not endocytosis at the immune synapse

## References

[1] Courtney AH, Lo WL, Weiss A (2018). TCR Signaling: Mechanisms of Initiation and Propagation. Trends Biochem Sci.

[2] Alcover A, Alarcon B, Di Bartolo V (2018). Cell Biology of T Cell Receptor Expression and Regulation. Annu Rev Immunol.

[3] Blanchard N (2002). TCR activation of human T cells induces the production of exosomes bearing the TCR/CD3/zeta complex. J Immunol.

[4] Choudhuri K (2014). Polarized release of T-cell-receptor-enriched microvesicles at the immunological synapse. Nature.

[5] Saliba DG (2019). Composition and structure of synaptic ectosomes exporting antigen receptor linked to functional CD40 ligand from helper T cells. Elife.

[6] Martell JD (2012). Engineered ascorbate peroxidase as a genetically encoded reporter for electron microscopy. Nat Biotechnol.

[7] Jenkins MR (2014). Distinct structural and catalytic roles for Zap70 in formation of the immunological synapse in CTL. Elife.

[8] Monks CR, Freiberg BA, Kupfer H, Sciaky N, Kupfer A (1998). Three-dimensional segregation of supramolecular activation clusters in T cells. Nature.

[9] Stein JM, Luzio JP (1991). Ectocytosis caused by sublytic autologous complement attack on human neutrophils. The sorting of endogenous plasma-membrane proteins and lipids into shed vesicles. Biochem J.

[10] Stinchcombe JC (2001). Rab27a is required for regulated secretion in cytotoxic T lymphocytes. J Cell Biol.

[11] Jenkins MR (2015). Failed CTL/NK cell killing and cytokine hypersecretion are directly linked through prolonged synapse time. J Exp Med.

[12] Gawden-Bone CM (2018). PIP5 Kinases Regulate Membrane Phosphoinositide and Actin Composition for Targeted Granule Secretion by Cytotoxic Lymphocytes. Immunity.

[13] Allan D, Thomas P, Michell RH (1978). Rapid transbilayer diffusion of 1,2-diacylglycerol and its relevance to control of membrane curvature. Nature.

[14] Allan D, Michell RH (1975). Accumulation of 1,2-diacylglycerol in the plasma membrane may lead to echinocyte transformation of erythrocytes. Nature.

[15] Carrasco S, Merida I (2004). Diacylglycerol-dependent binding recruits PKCtheta and RasGRP1 C1 domains to specific subcellular localizations in living T lymphocytes. Mol Biol Cell.

[16] Melowic HR (2007). Mechanism of diacylglycerol-induced membrane targeting and activation of protein kinase Ctheta. J Biol Chem.

[17] Ritter AT (2015). Actin depletion initiates events leading to granule secretion at the immunological synapse. Immunity.

[18] Kumari S (2020). Cytoskeletal tension actively sustains the migratory T-cell synaptic contact. EMBO J.

[19] Cantrell DA, Davies AA, Crumpton MJ (1985). Activators of protein kinase C down-regulate and phosphorylate the T3/T-cell antigen receptor complex of human T lymphocytes. Proc Natl Acad Sci U S A.

[20] Valitutti S, Muller S, Cella M, Padovan E, Lanzavecchia A (1995). Serial triggering of many T-cell receptors by a few peptide-MHC complexes. Nature.

[21] Valitutti S, Muller S, Dessing M, Lanzavecchia A (1996). Different responses are elicited in cytotoxic T lymphocytes by different levels of T cell receptor occupancy. J Exp Med.

[22] Valitutti S, Muller S, Dessing M, Lanzavecchia A (1996). Signal extinction and T cell repolarization in T helper cell-antigen-presenting cell conjugates. Eur J Immunol.

[23] Valitutti S, Muller S, Salio M, Lanzavecchia A (1997). Degradation of T cell receptor (TCR)-CD3-zeta complexes after antigenic stimulation. J Exp Med.

[24] Kelly BT (2014). Clathrin adaptors. AP2 controls clathrin polymerization with a membrane-activated switch. Science.

[25] De Franceschi N (2016). Selective integrin endocytosis is driven by interactions between the integrin alpha-chain and AP2. Nat Struct Mol Biol.

[26] Traub LM, Bonifacino JS (2013). Cargo recognition in clathrin-mediated endocytosis. Cold Spring Harb Perspect Biol.

[27] Szymczak AL, Vignali DA (2005). Plasticity and rigidity in adaptor protein-2-mediated internalization of the TCR:CD3 complex. J Immunol.

[28] Cao M (2015). Uni-directional ciliary membrane protein trafficking by a cytoplasmic retrograde IFT motor and ciliary ectosome shedding. Elife.

[29] Nager AR (2017). An Actin Network Dispatches Ciliary GPCRs into Extracellular Vesicles to Modulate Signaling. Cell.

[30] Phua SC (2017). Dynamic Remodeling of Membrane Composition Drives Cell Cycle through Primary Cilia Excision. Cell.

[31] Garcia G, Raleigh DR, Reiter JF (2018). How the Ciliary Membrane Is Organized Inside-Out to Communicate Outside-In. Curr Biol.

[32] Nachury MV, Mick DU (2019). Establishing and regulating the composition of cilia for signal transduction. Nat Rev Mol Cell Biol.

[33] Tolmachova T, Abrink M, Futter CE, Authi KS, Seabra MC (2007). Rab27b regulates number and secretion of platelet dense granules. Proc Natl Acad Sci U S A.

[34] Schuhmacher M (2020). Live-cell lipid biochemistry reveals a role of diacylglycerol sidechain composition for cellular lipid dynamics and protein affinities. Proc Natl Acad Sci U S A.

[35] von Schwedler UK (2003). The protein network of HIV budding. Cell.

[36] Schindelin J (2012). Fiji: an open-source platform for biological-image analysis. Nat Methods.

