# Supplementary material for: Ectocytosis renders T cell receptor signaling self-limiting at the immune synapse
Source: Science. Author manuscript; Available in PMC 2023 Jul 12. (PMC7614748; doi:10.1126/science.abp8933)
Supplement: Supplementary Materials [file EMS177470-supplement-Supplementary_Materials.pdf]

Science
AAAS

Supplementary Materials for

Ectocytosis renders TCR signaling self-limiting at the immune synapse

Jane C. Stinchcombe[1], Yukako Asano[1], Christopher J.G. Kaufman[1], Kristin Böhlig[2], Christopher J. Peddie[3], Lucy M. Collinson[3], André Nadler[2] and Gillian M. Griffiths[1*]
*Corresponding author: gg305@cam.ac.uk

The PDF file includes:

Materials and Methods
Figs. S1 to S11
References

Other Supplementary Materials for this manuscript include the following:

Movies S1 to S9

References (33-36)

Materials and Methods

Mice

Tg(TcraTcrb)1100Mjb Rag1$^{tm1Bal/tm1Bal}$ (referred to as OTI) mice were bred on a C57BL/6 background. *Rab27a*$^{ash/ash}$ *Rab27b*$^{-/-}$ mice (a kind gift from Miguel Seabra (33)) were crossed with OTI mice to achieve the OTI *Rab27a*$^{ash/ash}$ *Rab27b*$^{-/-}$ *Rag1*$^{tm1Bal/tm1Bal}$ strain (referred to as *Rab27a/b*$^{-/-}$ OTI). This research has been regulated under the Animals (Scientific Procedures) Act 1986 Amendment Regulations 2012 following ethical review by the University of Cambridge Animal Welfare and Ethical Review Body (AWERB).

Plasmids and antibodies

APEX-GFP constructs were prepared in the laboratory as previously described for EGFP-PKCγ-C1 (*12*). Engineered pea ascorbate peroxidase (APEX) was cloned from pcDNA3-mito-APEX, (Addgene plasmid # 42607) using primers 5'TTTTGGATCCACCATGGGAAAGTCTTACCCA ACTGTGAGTGC-3' and reverse 5'-AAAAGGATCCCCGGCATCAGCAAACCCAAGCTCG G-3' that added a 5' start codon and partial Kozac sequence, removed the stop codon and included terminal BamHI restriction sites, via a carrier plasmid pSC-B, into the BamHI site of EGFP-N1 (Clontech, Takara) to generate APEX-EGFP-N1. Lifeact-APEX-EGFP was constructed using 5' phosphorylated primers: 5'TCGAGGCCACCATGGGCGTGGCCGACTTGATCAAGAAGTTC GAGTCCATCTCCAAGGAGGAGGGG-3' (forward) and 5'GTACCCCCTCCTCCTTGGAGA TGGACTCGAACTTCTTGATCAAGTCGGCCACGCCCATGGTGGCC-3' (reverse) inserted into APEX-EGFP-N1 using XhoI and KpnI sites (ThermoFisher, # FD0694 and # FD0524). CD3ζ-APEX-EGFP was generated from a synthetic gene (Accession # NM_001113391.2; gBlock, Integrated DNA Technologies) with an XhoI restriction and the TAA stop codon replaced with a KpnI restriction site. This gBlock was subcloned via the carrier plasmid (as above) into the XhoI and KpnI sites of APEX-EGFP-N1. PKCε-EGFP (*34*) and EGFP-PKCγ-C1 (*12*) were as previously described; pmaxGFP (Lonza); dsRed-VPS4B E235Q (35) was a gift from Wes Sundquist and EGFP-VPS4B E235Q from Paul Luzio.

Primary and secondary antibodies were as follows:

**Antibody**	**Clone**	**Supplier**	**Cat. No.**	**RRID**
rat anti-CD8a	clone 53-6.7	ThermoFisher	14-0081-82	AB467087
rabbit anti-pZap70 (Tyr493)		ThermoFisher	PA5-17778	AB10983603
mouse anti-Zap70	clone1E7.2	ThermoFisher	37-6500	AB2533332
mouse anti-pCD3ζ (Tyr72)	clone EM26	ThermoFisher	MA5-28537	AB2745496
rabbit anti-CD3ε		Absolute Antibody	ab00121	
hamster anti-CD3ζ	clone H146-968	Abcam	ab119827	AB10902095
hamster anti-TCR β chain	clone H57-597	Biolegend	109201	AB313424
mouse anti-Granzyme B	clone GB11	Biolegend	515408	AB2562196
mouse anti-Lck	clone 3A5	Merck Millipore	05-435	AB309733
rat anti-CD44	clone IM7	BD Transduction Labs	550538	AB393732
mouse anti-PKC-θ	clone 27/PKC	BD Transduction Labs	610090	AB397497
mouse anti-Actin	clone AC-40	Sigma	A3853	AB262137
donkey-anti rabbit IgG(H+L)		Jackson Labs	711-545-152	AB2313584
goat anti-mouse IgG(H+L)		ThermoFisher	A-11029	AB2534088
goat anti-mouse IgG(H+L)		ThermoFisher	A-11031	AB144696
goat anti-mouse IgG(H+L)		ThermoFisher	A-21052	AB2535719
donkey anti-mouse IgG(H+L)		ThermoFisher	A-21202	AB141607
donkey anti-mouse IgG(H+L)		ThermoFisher	A-10036	AB2534012
goat anti-rabbit IgG(H+L)		ThermoFisher	A-11034	AB2576217
goat anti-rabbit IgG(H+L)		ThermoFisher	A-11035	AB2534093
donkey anti-rabbit IgG(H+L)		ThermoFisher	A-31572	AB162543
goat anti-hamster IgG(H+L)		ThermoFisher	A-21111	AB2535760
goat anti-hamster IgG(H+L)		ThermoFisher	A-21110	AB2535759
goat anti-rat IgG(H+L)		ThermoFisher	A-11077	AB2534121
goat anti-rat IgG(H+L)		ThermoFisher	A-11081	AB2534125
goat anti-rat IgG(H+L)		ThermoFisher	A-21094	AB2535749

Cell culture

CTLs were generated by stimulating splenocytes derived from WT OTI or *Rab27a/b*$^{-/-}$ OTI mice with 10 nM SIINFEKL (Cambridge Bioscience) for 3 days in RPMI 1640 medium (Sigma-Aldrich, # 1640) with 10% FBS (LabTech, # FBS-SA), 50 mM β-mercaptoethanol (ThermoFisher, # 31350010), 10 U/ml recombinant murine IL-2 (Peprotech, # 212-12), 2 mM L-Glutamine (Sigma-Aldrich, # G7513-100ML), 1 mM sodium pyruvate (ThermoFisher Scientific, # 11360070) and 50 U/ml penicillin and streptomycin (Sigma-Aldrich, # P0781-100ML). Cells were spun down and seeded into fresh media daily from 3 days post stimulation. Target EL4 cells

(ATCC-TIB-39; RRID: CVCL_0255) were maintained in DMEM (Sigma-Aldrich, # D5030-10X1L) supplemented with 10% FBS and 2 mM L-glutamine. For light microscopy experiments, EL4 target cells expressing the blue inner leaflet plasma membrane marker, Farnesyl-5-TagBFP2 (EL4-blue) were used (*17*).

Nucleofection

Plasmids were nucleofected into OTI CTLs 4-6 days after activation. Briefly, 5-10 x 10^6 activated OTI cells were washed in PBS and nucleofected with 2.5 µg cDNA in 100 µl nucleofection solution from the mouse T Cell Nucleofector Kit (Lonza) using the DN-100 program according to the manufacturer's instructions. Nucleofected cells were plated in 1 ml nucleofection medium (Lonza), incubated for 1-2 hours to recover, then diluted to a final volume of 12-16 ml with culture media. 7 µM haem was added to the media for nucleofections with APEX-containing constructs for samples processed in ultrastructural studies.

Conjugate formation and processing for ultrastructural studies

16-24 hours after nucleofection, OTI CTLs expressing APEX constructs were washed twice in culture serum-free medium and resuspended at 1-2 x 10^6 per ml. CTLs were plated onto tissue culture plates (Nunc) for TEM or coverslips marked with grids (Bellco Glass) for FIB-SEM, either alone or after mixing 1:1 with EL4 target cells pulsed with SIINFEKL peptide. CTLs and targets were allowed to form conjugates for 20, 40 or 60 min at 37°C after which they were fixed in pre-warmed fixative containing 2% PFA (Electron Microscopy Sciences) and 1.5% glutaraldehyde (Agar Scientific) for 20 min at RT, then 10-30 min at 4°C. This allowed us to capture CTLs at different stages of immune synapse formation as CTLs engaged with targets at varying time points up to the point of fixation. Thus, 20, 40 and 60 min samples contain conjugates formed from <1 min to a maximum of 20, 40 or 60 min, respectively. The samples were maintained at 4°C for all subsequent steps until dehydration to minimise diffusion of the cytochemical reaction product. Fixed samples were washed in 0.1 M sodium cacodylate, pH 7.4, rinsed in Tris Buffer (0.5 M Tris-HCl, pH 7.6), and processed for Diaminobenzidene (DAB) cytochemisty by incubating for 15 min in DAB reaction mixture (DAB (Sigma) (0.75 mg/ml), 0.21% (v/v) hydrogen peroxide in Tris buffer) in the dark. Samples were washed in Tris Buffer followed by 0.1 M sodium cacodylate, pH 7.4 then post-fixed in 0.1% osmium tetroxide (TAAB) for 1 hour followed by 0.5% aqueous

uranyl acetate (TAAB) overnight, washing with deionised H_2O in between. For TEM, post-fixed samples were dehydrated in increasing concentrations of ethanol (from 50%-100%) and embedded in EPON resin (Agar Scientific). For FIB-SEM, samples were further post-fixed for 1 hour in lead aspartate (0.066 g of lead nitrate in 10 ml of 0.03 M aspartic acid solution pH 5.5), washed in deionised H_2O then dehydrated in increasing concentrations of ethanol (50-100%) followed by 100% acetone, and the whole coverslip embedded with Durcupan (Sigma). Untransfected CTLs were used 5-6 days after activation and prepared as above for TEM, except samples were not moved to 4°C for processing and the DAB-cytochemistry step was omitted.

For conventional TEM imaging, thin (50-70 nm) and semi-thick (100-200 nm) sections of samples were collected on grids, analyzed +/- lead citrate staining using an FEI Technai Spirit TEM (FEI) and images captured using a Gatan camera. Images were false colored to distinguish target cells (in blue) using Adobe Photoshop. >125 or 683 immune synapses and >54 or 50 single CTLs from across five (Lifeact-APEX-EGFP) and five (CD3ζ-APEX-EGFP) independent nucleofection experiments, respectively, and >50 immune synapses from seven separate untransfected cell preparations, were analyzed for this study.

For TEM tomography, 250-300 nm serial sections across the immune synapses of 12 cells were collected on formvar-coated slot grids, and the top and bottom of the sections decorated with 15 nm fiducial gold (British Biocell International). Tilt series images of cells of interest were collected manually for each serial section at 2° intervals from -60° to +60° using the FEI Technai Spirit TEM (FEI, Eindhoven) with Gatan camera, as above. Four consecutive tilt series were collected for each of the tomograms shown in Fig. 2 and Fig. 3, E to F. Five consecutive tilt series were collected for the tomogram shown in Fig. 3, A to D. Tilt images were converted into ".mrc" series files using Cygwyn software and tomograms generated and serial tomograms joined together using the IMOD and 3dMOD packages (Boulder Laboratory for 3-D EM of cells, University of Colorado, USA). Models of the joined tomograms were generated using 3dMOD program (Boulder). Image J software was used for additional image processing and movie generation.

To quantitate the distribution of CD3ζ-APEX label in CTL-target conjugates at different time points, thin sections were prepared from samples of cells fixed 20, 40 or 60 min after cell mixing

from four independent experiments. Images were captured of every conjugate associated with any APEX labeling within each single thin section for each fixation time point. 107 APEX-labeled conjugate images were captured from 20 min samples, 146 for 40 min and 49 for 60 min. Each image was examined for the presence or absence of APEX label in projections, flattened projections, cSMAC or ectosomes and the percentage of conjugates with APEX in a particular structure was calculated for each structure for each time point. Only the presence or absence of label in a structural type in a conjugate was being measured, not the number of labeled structures per conjugate. Note that a stronger signal may arise from the APEX reaction product becoming concentrated within curved tips or vesicles. Graphs throughout this study were generated using Prism software with error bars showing the mean with standard deviation (SD).

To quantitate the association of ectosomes and buds with CTL and/or target membranes at the immune synapse in the reconstructed tomogram shown in Fig. 3 A to D, each identified ectosome or budding ectosome present between the CTL and target at the contact site was followed through the reconstructed tomogram section series and any association with the CTL surface membrane and/or the target surface membrane, recorded. The number of ectosomes or buds in contact with each cell surface was expressed as a percentage of the total ectosome (293) or bud (29) number. The area of CTL immune synapse membrane labeled with the APEX probe was calculated by capturing .tif images of reconstructed tomogram models (Figs. 2 and 3) face on across the immune synapse and using the lasso tool in Adobe Photoshop to calculate the number of pixels occupied by labeling in these images, and thus the total labeled surface area.

FIB SEM data was collected using a Crossbeam 540 FIB SEM with Atlas 5 for 3-dimensional tomography acquisition (Zeiss). A segment of the cell monolayer containing the conjugates of interest was located using grid coordinates, trimmed, and the coverslip removed using liquid nitrogen. The sample was mounted on a standard 12.7 mm SEM stub using silver paint and coated with a 5 nm layer of platinum. The target area was relocated by briefly imaging through the platinum coating at an accelerating voltage of 10 kV and correlating to previously acquired images. On completion of preparation of the target ROI for Atlas-based milling and tracking, images were acquired at 5 nm isotropic resolution throughout the cells of interest, using a 10 μs dwell time. During acquisition the SEM was operated at an accelerating voltage of 1.5 kV with 1.5 nA current.

The EsB detector was used with a grid voltage of 1,200 V. Ion beam milling was performed at an accelerating voltage of 30 kV and current of 700 pA. After acquisition, the images were batch processed to suppress noise and enhance sharpness/contrast (i. gaussian blur 0.7-pixel radius; ii. smart sharpening with highlights suppressed: radius 10 pixels, strength 100%, iii. grey levels adjustment to enhance contrast, iv. convert to 8-bit greyscale; Adobe Photoshop). The processed dataset was then aligned using the register virtual stack slices plugin in Fiji (*36*).

Immunofluorescence

EL4 target cells alone or OTI CTL-target conjugates formed as described above for EM were plated onto either multi-well slides (for confocal analysis) or pre-cleaned glass coverslips (for Elyra SIM analysis) and incubated at 37°C for 25-45 min. Cells were either fixed and permeabilized with -20°C methanol for 5 min or fixed with 4% PFA in PBS, quenched with 15 mM glycine in PBS (20 min) and permeabilized with 0.05-0.2% Triton-X100 (5 min). Fixed samples were blocked in 2% BSA in PBS at least 15 min before incubation for 1 hour at room temperature or overnight at 4°C with primary antibodies, then 1 hour with Alexa 488-, 568- or 633-conjugated secondary antibodies, all cross-absorbed against relevant species. Some samples were incubated 5 min with Hoechst (1:25,000 in PBS) to label nuclei. Samples were washed extensively between each incubation and mounted with Vectashield (Vector Laboratories Inc.) for confocal microscopy or ProLong gold (ThermoFisher), and mounted in accordance with Zeiss guidelines, for SIM. 2-3 replicates were prepared per condition per experiment.

For experiments varying TCR signal strengths, equal numbers of EL4 target cells were pulsed with either 1 μM irrelevant NP68 peptide, 1 μM "weak" SIIGFEKL (G4) peptide or "strong" SIINFEKL (N4) peptides (Cambridge Biosciences) at 1 nM, 10 nM, 100 nM or 1 μM. EL4 samples were washed free of unbound peptide identically, resuspended to the same final volumes, and mixed with an equal volume of CTLs from a single suspension (final ratio of 1.5 to 1), pipetting up and down in between to prevent settling and ensure an identical sample of CTLs was delivered to each EL4 sample. Each sample was mixed by pipetting up and down and distributed in 50 μl aliquots across multi-well slides. Slides were incubated at 37°C for 35 min then fixed and processed together for immunofluorescence as above, treating all samples identically and using the same preparation of antibodies across all samples for each labeling condition. For experiments

comparing OTI and *Rab27a/b*$^{-/-}$ OTI CTLs, CTLs were mixed with 1 μM SIINFEKL pulsed-EL4 targets, incubated at 37°C for 40 min then fixed and processed as above.

For confocal images, samples were analyzed using an Andor spinning-disk confocal system (Revolution; Andor) fitted with a spinning-disk unit (CSU-X1; Yokogawa) and used with lasers exciting at 405, 488, 543 and 633 nm. Samples were viewed with either an Olympus IX81 microscope, using an Olympus 100x objective, numerical aperture 1.40, or a Leica DMi8 microscope, using Leica 40x (for quantitation images) or 100x objectives with numerical apertures of 1.3 (40x), 1.4 or 1.44 (100x). Z-stack image series through cells of interest were collected at 0.07-0.1 μm intervals across the depth of the cell and captured using a iXon Ultra 888 camera and either IQ3 or Fusion software (Andor). Images of EL4 cells alone labeled with ectosome markers were collected and subsequently processed using identical settings as those used for conjugates. Images for comparative analysis were collected with identical laser settings across all samples for each antibody condition using either a 100x lens or, for quantitative analysis, the 40x lens. 4-8 fields (depending on experiment and cell density) were collected with identical settings per condition per antibody combination for quantitation. For SIM experiments, preliminary data on antigens of interest was first established using confocal microscopy experiments and new samples were then prepared for higher resolution SIM analysis. SIM images were collected on the Zeiss Elyra PS1 and, using the Zeiss Zen Black software, were processed, and aligned using an affine method with corrections derived by imaging multispectral sub resolution beads. Imaris software (Bitplane) was used to analyse stacks and obtain single plane images and/or projection images of 3D reconstructions for both confocal and SIM data. In some images (figs. S5 and S6), the blue EL4 membrane is false colored white, and far red false colored blue. Final images were processed with Adobe Photoshop (CS4-CS6; Adobe) software. >8767 immune synapses were analyzed for this study across four (CD3ζ-APEX-EGFP), five (EGFP-PKCγ-C1), two (EGFP-PKCε), three (dsRed-VPS4B E235Q, EGFP-VPS4B E235Q and pmaxGFP), three (Rab27a/b-deficient), more than five (signaling peptide) or >22 (untransfected, untreated) independent experiments. Representative SIM and confocal images are shown.

Quantitative analysis of immunofluorescence experiments.

For quantitative analysis of cells incubated under different peptide-activation conditions samples were labeled with antibodies against CD3ε and either PKCθ, Zap70 or Lck, and imaged using a 40x lens and identical laser settings. 4-8 whole field images were collected for each condition across replicates. Images were viewed in Imaris and the number of OTI CTL-EL4-blue conjugates (defined by immune synapse accumulated PKCθ, Zap70 or Lck), targets with transferred CD3ε-labeled ectosomes, and CTLs with CD3ε surface expression were counted per field. Quantitation was carried out using identical intensity threshold values, determined by settings which gave non-saturating, specific labeling in positive control samples (1 μM N4) but no background signal on EL4 cells alone and/or when using the irrelevant peptide, NP68. These threshold settings mean some low intensity labeling, particularly of ectosomes and at the immune synapse, may not have been detected. For cell loss over 35 min at 37°C with different signal strength, the number of CTLs present in each activated sample field was expressed as a percentage of the mean of the non-activating (NP68) sample CTL number. For CD3ε surface labeling, the number of CTLs with CD3ε surface labeling is expressed as a percentage of total CTLs in the same field. For ectosome transfer, the number of targets per field decorated with CD3ε ectosomes are expressed as a percentage of total targets in each field. Three independent quantitation experiments using CTLs prepared from three different spleens were performed. Data from one experiment, representative of all three, is shown, with each data point representing one image field. 163-358 CTLs and 274-463 targets were analyzed per condition, depending on condition and experiment. For samples comparing OTI and Rab27a/b-deficient OTI CTLs, four fields with ≥194 CTLs were captured and ectosomes analyzed as above using the same thresholds and conditions. Data from one experiment representative of three independent experiments is shown, with n = 1022 OTI and n = 1265 Rab27a/b-deficient OTI.

For quantitation of samples containing CTLs expressing dsRed-VPS4B E235Q, EGFP-VPS4B E235Q or pmaxGFP as control, mixed with targets and fixed 40 min after incubation at 37°C, 4-11 fields were collected per condition and analyzed using Imaris as above. For each field, the number of CTLs expressing construct (+), defined by presence of fluorescent tag, and CTLs showing no expression (-) were counted, along with the number of expressing (+) and non-expressing (-) CTLs showing any contact with targets. CTLs in contact with targets were analyzed

for accumulation of PKCθ, Lck or Zap70 signaling proteins at any contact and for the presence of CD3ε-ectosomes on associated targets as above, with intensity threshold levels determined from the signal accumulation and transferred ectosome labeling given by non-expressing cells within the same samples. For each expressing (+) or non-expressing (-) population, the number of CTLs with immune synapse accumulation or ectosome transfer was expressed as a percentage of the total CTLs in each sample, as appropriate. Data from two separate experiments, representative of three biologically independent experiments is shown. A total of 726 EGFP-VPS4B E235Q-, 976 dsRed-VPS4B E235Q-, and 472 pmaxGFP- expressing CTLs were analyzed.

For quantitation of Zap70 distribution, 40x images of conjugates incubated for 35-45 min at 37°C and labeled with CD3ε and Zap70 were collected from replicates across three independent experiments and analyzed using Imaris as above. For each experiment, images were assayed for the number of Zap70-positive conjugates with Zap70 at the immune synapse (either cSMAC or ectosome labeling), transferred to ectosomes on targets, in clusters at the CTL surface or inside the CTL. Each value was expressed as percentage of the total number of Zap70-positive conjugates for each of the three independent experiments (n = 50, 56, 120).

To quantitate ectosome content, images were collected of untransfected CTLs or CTLs expressing CD3ζ-APEX-EGFP or DAG bio-probes, fixed after 35-40 min incubation at 37°C with targets and co-labeled for CD3ε and either CD8, TCRβ, Zap70, PKCθ, Lck, or GrB. For each marker, each immune synapse was assayed for the presence of labeling in CD3ε ectosomes using intensity thresholds as determined above. At least 1034, 158, 152, 180, 492, 144, 374, 289 and 114 immune synapses were analysed for CD3ε-, CD3ζ-, TCRβ-, CD8-, Zap70-, DAG-, PKCθ-, Lck-, or GrB-labeled samples, respectively. It should be noted that PKCθ is only visible when the signal at the immune synapse is very over-exposed and so the number of co-labeled targets is an underestimation. Data is expressed as the percentage of total immune synapses where label was detected in CD3ε ectosomes for each marker. To quantitate pZap70 in ectosomes, images were collected of conjugates prepared using untransfected CTLs as above and co-labeled for pZap70 and either Zap70 or CD8. Immune synapses showing Zap70 or CD8 in ectosomes (n = 40) were assayed for pZap70 co-localization, and the number of immune synapses with pZap70 in Zap70/CD8 ectosomes expressed as a percentage of total immune synapses with Zap70/CD8-

labeled ectosomes. For both analyses, images were collected from at least three independent experiments per marker. Each data point represents a separate experiment.

Live cell imaging

35 mm MatTek imaging petri dishes with 1.5 thickness coverslip glass-bottoms were coated with 0.5 µg/ml ICAM-I in PBS at 4°C overnight. EL4-blue target cells were allowed to attach to the glass for 5 min in serum-free medium after which imaging buffer (RPMI without phenol red, 10% FBS, 25 mM HEPES, 2 mM L-glutamine, 50 U/ml penicillin and streptomycin) was added to the dishes. Just before imaging CTLs expressing PKCε-EGFP (nucleofected 24 hours earlier) were resuspended in imaging buffer and added to the target cells drop-wise. After 5 min CTL and target conjugates were imaged using a spinning disk confocal microscope system (Andor) with CSU-X1 Spinning disc unit (Yokogawa) and 100x objective (Leica HC PL APO 100x/1.40 OIL CS2). The samples were excited with 405 and 488 nm lasers with Borealis Enhanced illumination and maintained at 37°C, 5% CO_2 in an air flow sample chamber on the stage (OkoLab) during imaging. Images were captured with an EMCCD iXon Ultra 888 camera with Fusion software (Andor). Z-stack images were taken every 0.3 µm over 10 µm, at 10-20 s intervals for about 15 min.

For uncaging experiments, OTI CTLs expressing PKCε-EGFP were allowed to settle on the glass coverslip of a MatTek imaging dish. Cells were incubated with 602 µM cgSAG, 0.05% Pluoronic F-127 (Invitrogen) as previously described (34) in imaging buffer without FBS at RT for 5 min, after which the solution was gently replaced with 2 ml imaging buffer without FBS and cells were imaged within 30 min after loading. Cells were first imaged before uncaging, using the 488 nm laser and a z-interval of 0.5 µm and 25 planes. 3 time points were acquired every 5 s. To uncage cgSAG and generate DAG, cells were exposed to the 405 nm laser at 100% power for 13 ms (z: interval 1 µm, 13 planes). 10 s after uncaging cells were imaged using the 488 nm laser (z: interval 0.5 µm, 25 planes) every 5 s. Imaris v7.7.2 (Bitplane) was used to generate both the figure and movie.

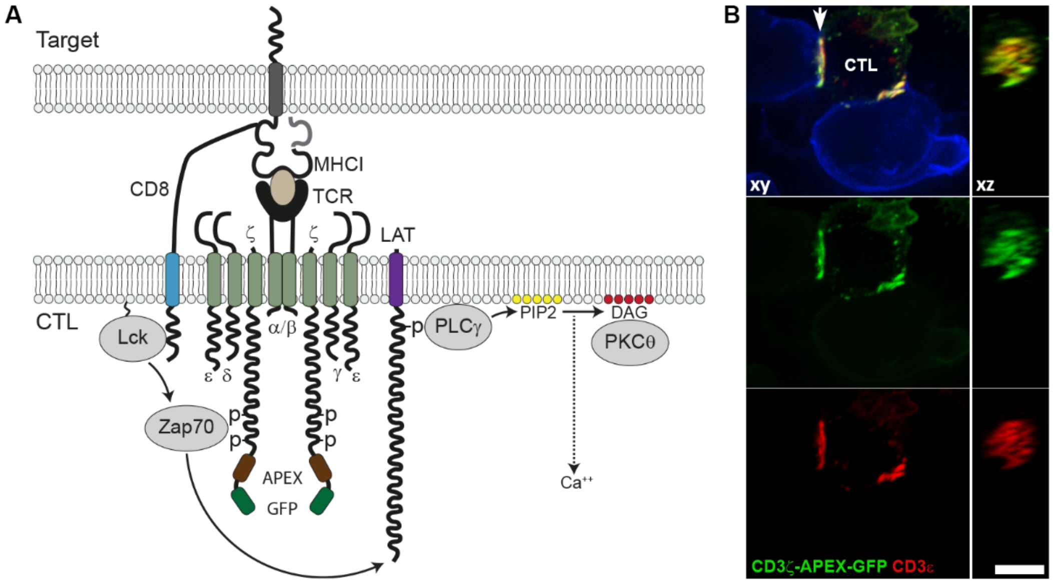

Fig. S1.

CD3ζ-APEX-EGFP co-localizes with endogenous CD3ε.

(**A**) Cartoon showing the TCR-CD3 complex with α and β chains involved in antigen recognition and CD3 δ, ε, γ and ζ chains required for signal transduction, illustrating the site of APEX and GFP tagging of CD3ζ. Kinases (Lck, Zap70 and PKCθ) described in this study are also shown with arrows pointing to pertinent targets of phosphorylation. In brief, TCR engagement with peptide (light brown) bound to MHCI triggers recruitment of Lck associated with CD8 leading to the phosphorylation of CD3ζ and Zap70 that phosphorylates Linker of T cell activation (LAT). LAT phosphorylation recruits PLCγ that cleaves PIP2 to generate DAG during T cell activation. (**B**) Projection (xy) and en face (xz) confocal images showing immune synapses (e.g. white arrow) between a CTL expressing CD3ζ-APEX-GFP (green) and two targets (blue), co-stained with antibodies to CD3ε (red). Image is representative of 105 immune synapses with markers as shown from four independent experiments. Scale bar, 2 μm.

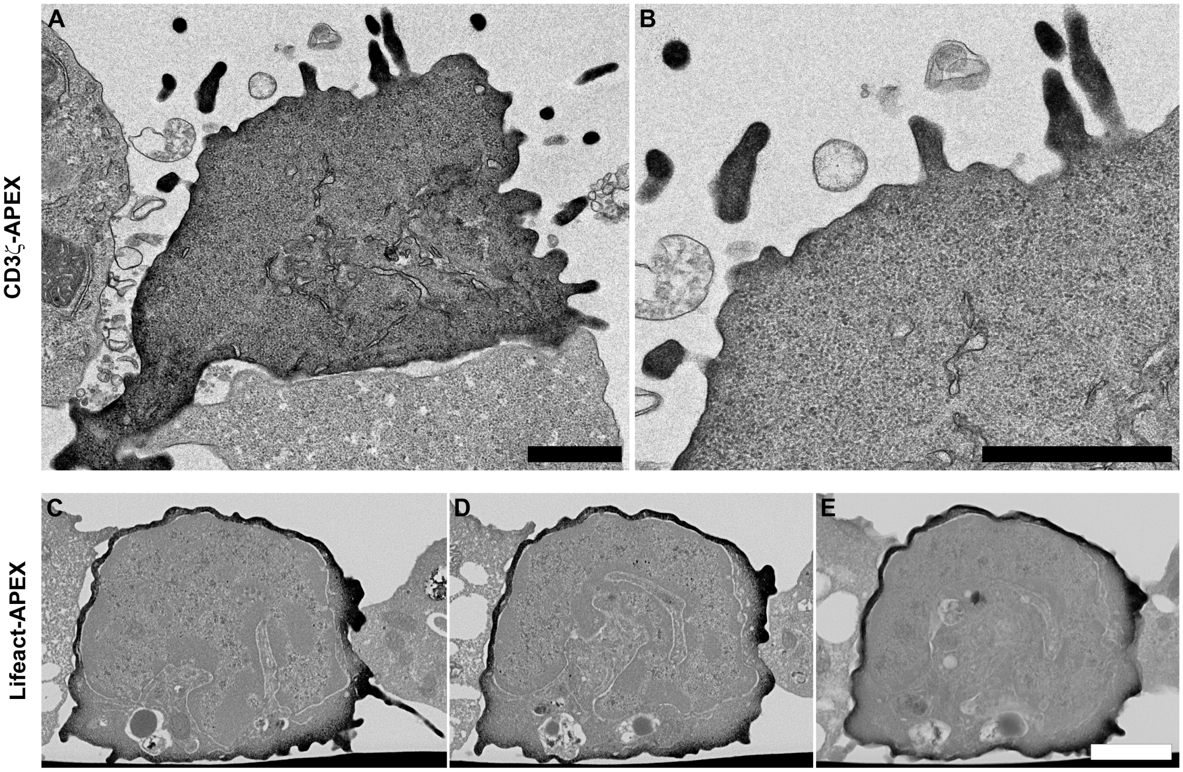

Fig. S2.

TEM and FIB-SEM imaging of CD3ζ-APEX and Lifeact-APEX in CTLs.

(**A** to **B**) TEM thin section (50-70 nm) image of a CTL expressing CD3ζ-APEX in the absence of target cells. Note only one CTL is expressing CD3ζ-APEX. Image is representative of 24 cells from four independent experiments. Scale bars, 1 μm. (**C** and **D**) Single and (**E**) projection images from a FIB-SEM sequence (movie S1) showing a CTL expressing Lifeact-APEX interacting with two target cells. The target on the left is dying (indicated by the vacuolated ER). Scale bar, 2 μm.

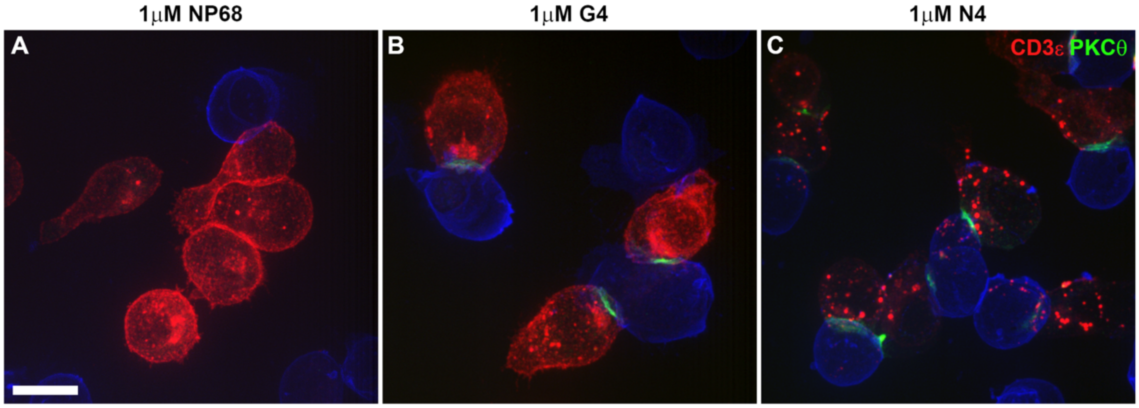

Fig. S3.

Increased TCR activation leads to decreased surface CD3ε and increased ectosome formation (quantitation shown in Fig. 4, B and C).

Representative confocal projection images corresponding to Fig. 4, B and C showing untransfected OTI CTLs incubated for 35 min with blue EL4 targets pre-loaded with 1 μM (**A**) NP68, (**B**) G4 or (**C**) N4 peptide, with CD3ε (red) and PKCθ to mark the synapse (green). For quantitation CTLs were counted as having surface CD3ε as illustrated in panels A (NP68) and B (G4) and no surface CD3ε as illustrated in panel C (N4). Images are representative of 163-358 CTLs and 274-463 targets per condition from replicate samples across more than five biologically independent experiments. Scale bar, 10 μm.

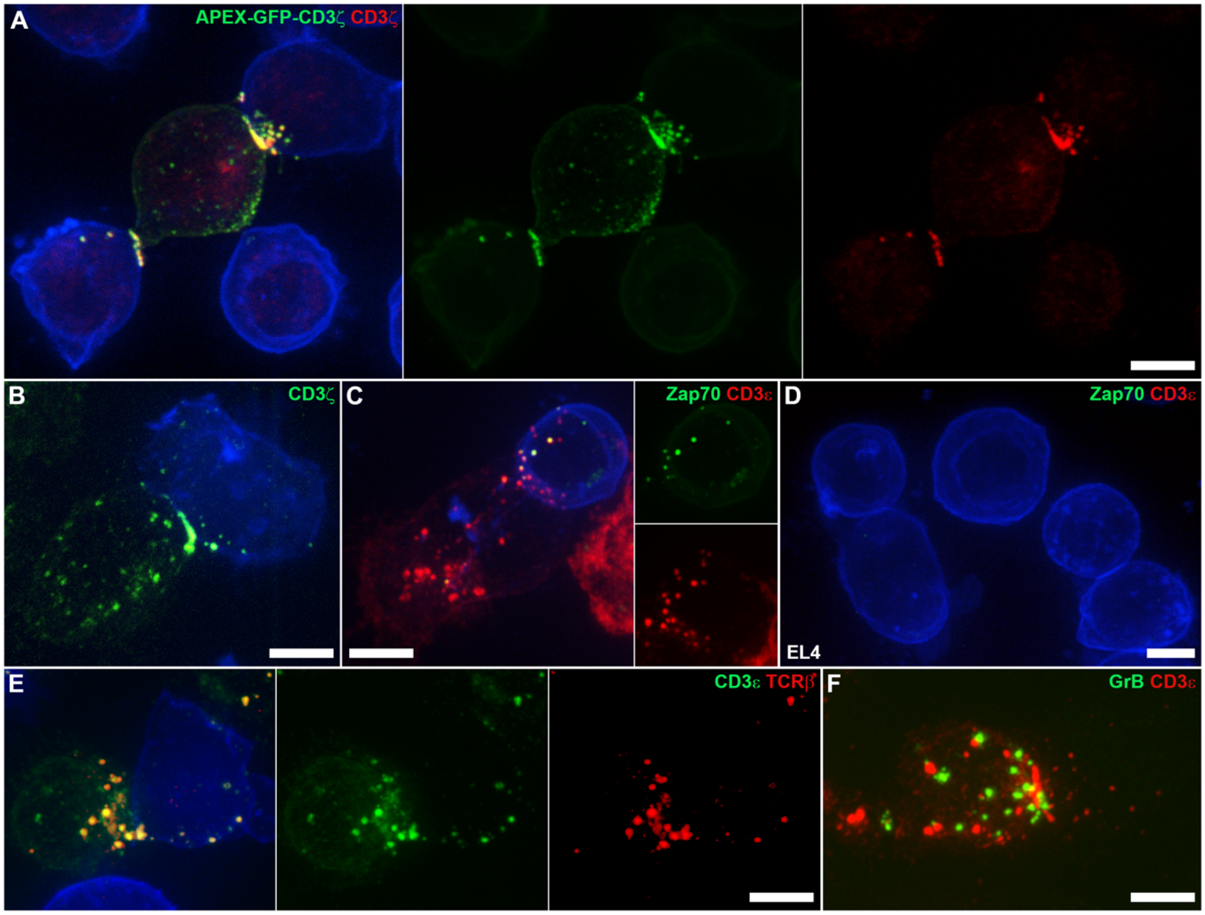

Fig. S4.

Endogenous proteins are transferred to target cells in ectosomes (quantitation shown in Fig. 5D).

(**A**) CTL expressing CD3ζ-APEX conjugated to targets (blue), labeled with anti-CD3ζ. (**B** and **C**) untransfected CTLs conjugated to targets (blue), or (**D**) target cells alone (EL4), stained with anti-CD3ζ, -CD3ε and -Zap70 as indicated. (**E** and **F**) untransfected OTI CTLs conjugated to targets (blue), labeled with antibodies against CD3ε and (E) TCRβ or (F) granzyme B (GrB) as indicated. Images are confocal projection images, representative of (A) 48; (B) 102; (C) 253; (D) 427; (E) 194; (F) 94 ectosome-labeled immune synapses with markers as shown from at least three to four independent experiments each. Scale bars, 5 μm.

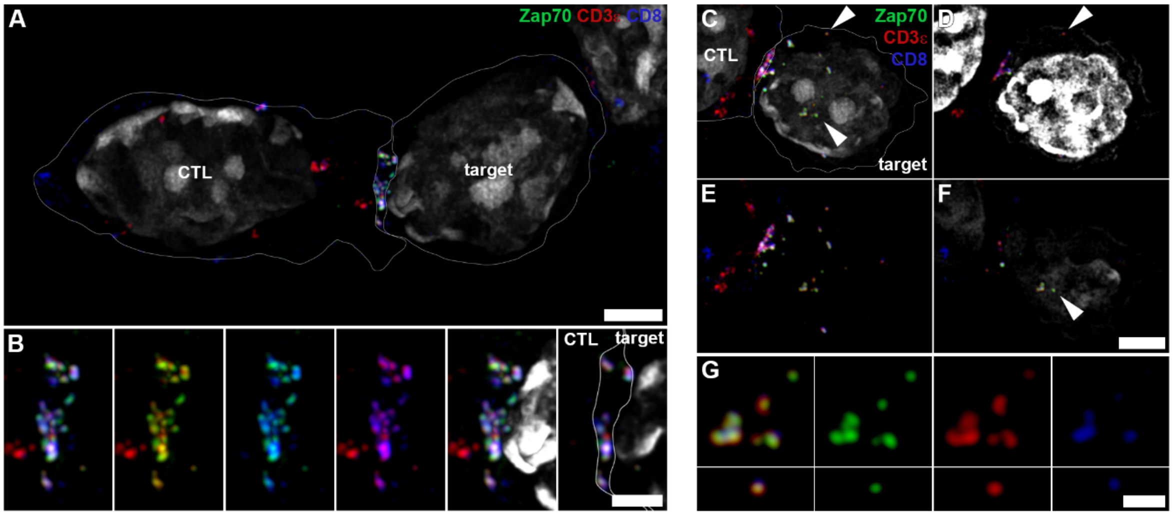

Fig. S5.

Endogenous CTL ectosomes labeling with CD3ε, CD8 and Zap70 are internalised into target cells (quantitation shown in Fig. 5D).

(**A** to **G**) SIM images of untransfected CTLs conjugated to targets, showing endogenous CD3ε (red), Zap70 (green) and CD8 (blue) in ectosomes, with Hoechst nuclear labeling (strong white label). Ectosomes at the immune synapse of (A) are shown in higher power in (B). White arrowheads (C, D and F) indicate ectosomes internalised into targets (white membrane, traced in (C) and enhanced in (D)), and shown at higher power in (G). (A to C, E and G) are projection and (D and F) single plane images. Images are representative of markers indicated from (A and B) 72 and (C to G) 69 immune synapses from five independent experiments. Scale bars, (A and F), 2 μm; (B), 1 μm; (G), 500 nm.

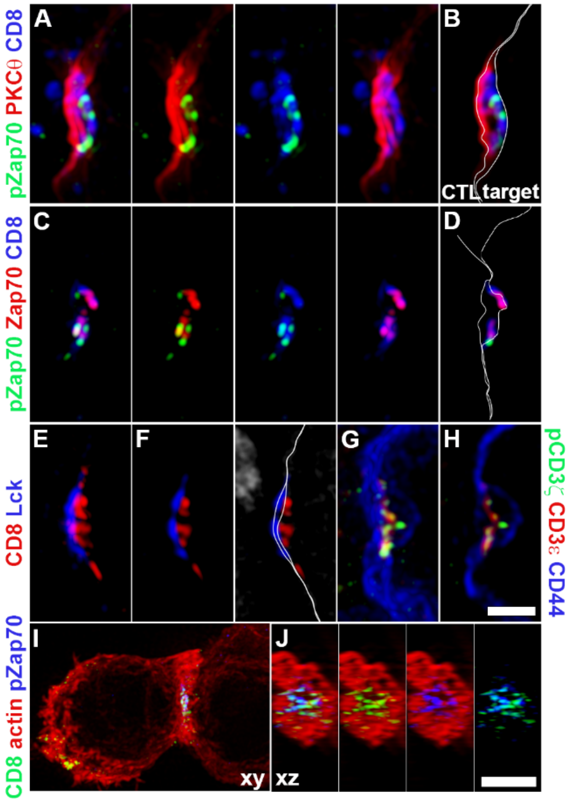

Fig. S6.

Ectosomes contain distinct signaling content (quantitation in Fig. 5, D and E).

(**A** to **H**) Expanded version of Fig. 5A showing projection (A and C and E and G) or single plane (B and D and F and H) SIM images of the immune synapse formed between untransfected CTL (left cell in each panel) and target (right cell in each panel), labeled with markers as indicated. Membranes are shown traced in white except for G and H where both cell membranes are labeled with CD44 (blue). (**I** and **J**). SIM images of a CTL conjugated to target, labeled as indicated, and shown either from the side (xy) as a full projection image (I) or en face (xy) across the immune synapse (J). All are representative images of markers as shown from (A and B) 17; (C and D) 45; (E and F) 98; (G and H) 25; (I and J) 12, immune synapses from three to five independent experiments each. Scale bars, (A to H) 1 μm, (I to J) 3 μm.

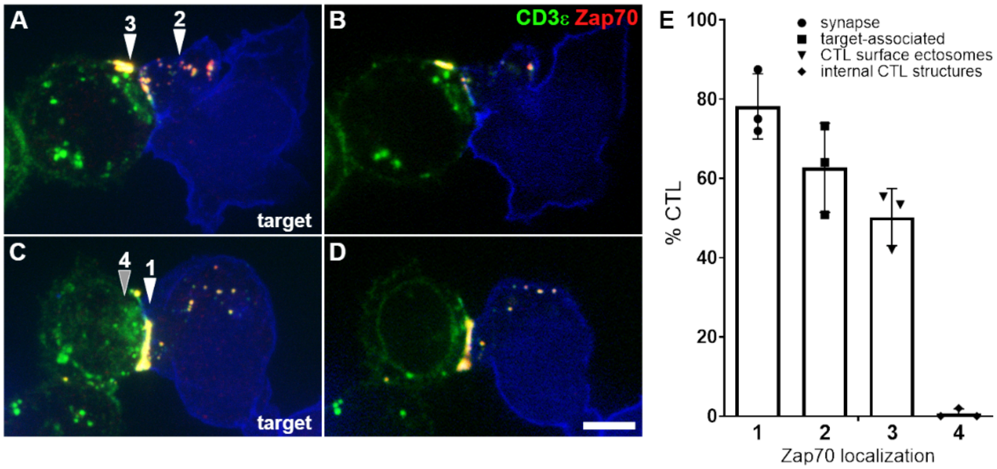

Fig. S7.

Zap70 is shed from the immune synapse with CD3ε in ectosomes but is not present in internal structures of the CTL.

Analysis of Zap70 distribution in CTL-target conjugates after 40-45 min incubation. (**A** to **D**) Confocal projection (A and C) and single plane (B and D) images of the immune synapse showing Zap70 (red) and CD3ε (green) co-labeling. Arrowheads show co-labeling (1) at the membrane and in ectosomes at the immune synapse; (2) in ectosomes transferred to targets; (3) at the CTL plasma membrane; but (4) not inside the CTL. Images are representative of >253 immune synapses with markers as shown across more than four independent experiments. Scale bar, 5 μm. (**E**) Quantitation of confocal images, as above, showing the percentage with Zap70 at each location indicated. Data from 223 CTLs is included. Error bars show mean values ± SD with each data point representing a biologically independent experiment (n = 3).

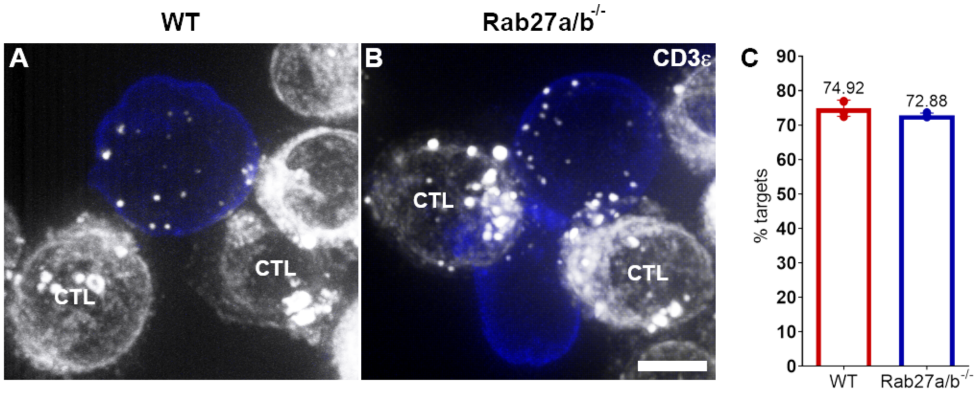

Fig. S8.

Ectocytosis is independent of cytolytic granule secretion.

Analysis of CD3ε-ectosome production at immune synapses formed between WT OTI CTLs or *Rab27a/b*$^{-/-}$ OTI, and targets (blue). (**A** and **B**) Representative confocal projection images showing CD3ε labeling (white) to identify ectosomes. (**C**) Quantitation of confocal images showing percentage of total targets with transferred CD3ε-ectosomes for WT (red) and *Rab27a/b*$^{-/-}$ (blue) samples. n = 1022 WT OTI and 1265 *Rab27a/b*$^{-/-}$ OTI CTLs analyzed. Data representative of three independent experiments. Error bars show mean values ± SD, with each data point representing one image field. Scale bar, 5 μm.

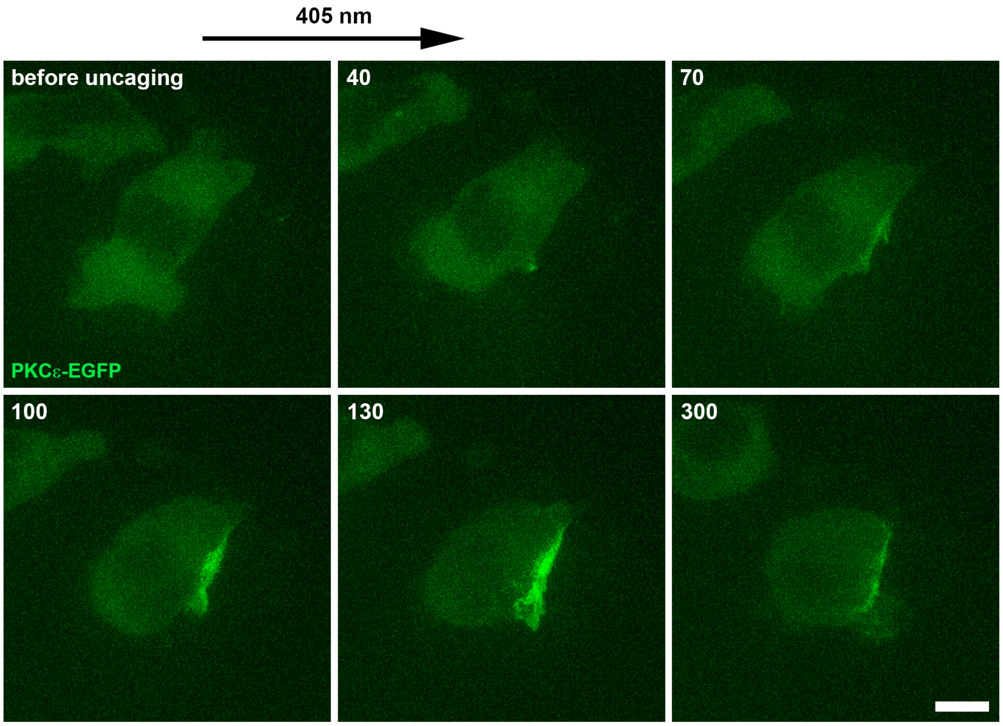

Fig. S9.

Specific binding of PKCε-EGFP to DAG.

Caged DAG (cgSAG) was used to confirm the specificity of PKCε-EGFP for DAG using live imaging (movie S8). . Images show the CTL before uncaging and at times indicated (s) after uncaging of cgSAG with 405 nm laser exposure. PKCε-EGFP detected DAG localized at an area of ruffling plasma membrane as shown. sImages are projections of 25 z-planes and are representative of >30 cells from 10 independent experiments. Scale bar, 5 µm.

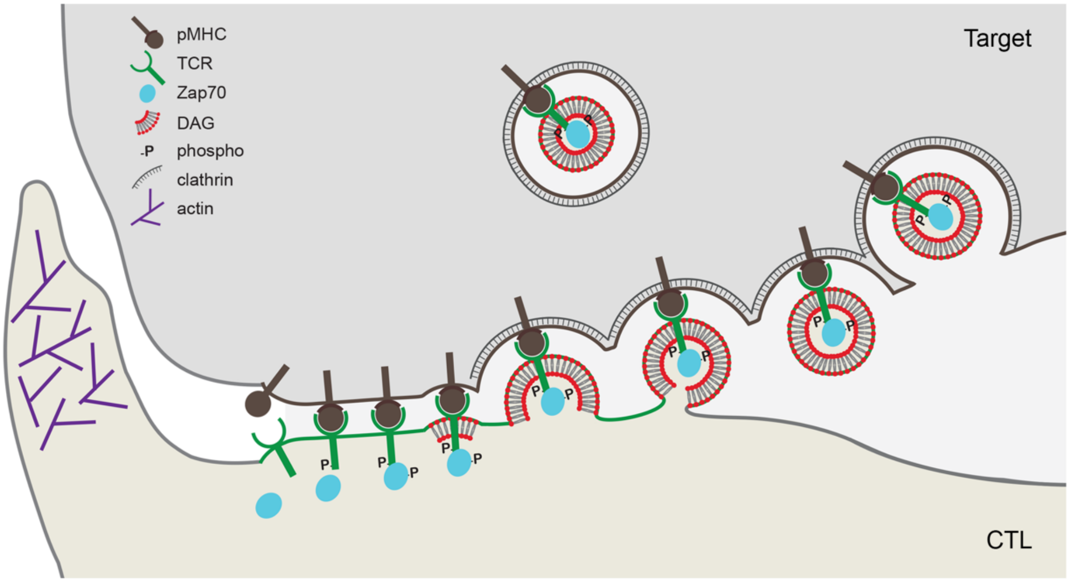

Fig. S10.

Cartoon showing model of TCR ectocytosis across the immune synapse.
TCR (green) is phosphorylated (p) after engagement with pMHC (brown), recruiting Zap70 (turquoise) and initiating accumulation of DAG (red) at the site of signaling. The negative membrane curvature generated by DAG causes outward membrane budding where receptor activation takes place. TCR and pMHC remain engaged as ectosomes bud from the CTL, causing localized detachment of CTL from target where TCR signaling has occurred. Ectosomes are internalized into targets via clathrin coated structures (black), terminating signaling from shed TCRs.

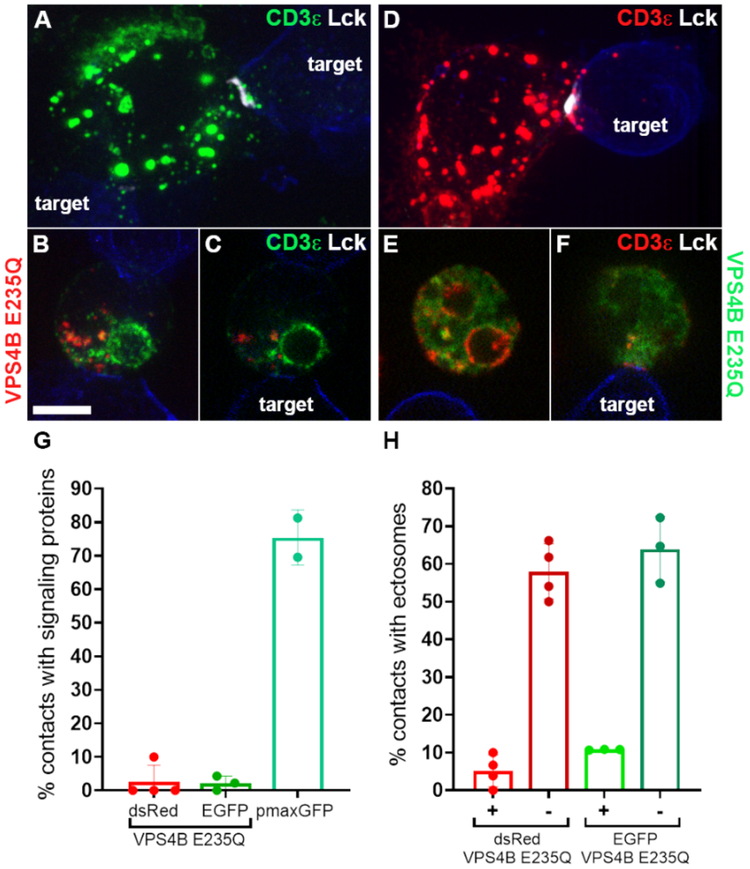

Fig. S11.

Inhibition of ESCRT VPS4 disrupts immune synapse formation.

(**A** to **F**) confocal images of CTLs conjugated to targets and co-labeled with antibodies to CD3ε and Lck as indicated. (A and D) show untransfected CTLs. (B to C, and E to F) show CTLs expressing dominant negative (dn) VPS4B E235Q tagged with dsRed or EGFP as indicated. CTLs expressing dnVPS4 showed disrupted trafficking of TCRs that localized to swollen endocytic compartments. Consequently, signaling proteins were not recruited to contact sites and immune synapses did not form. Images are (A and D) projections, (B) partial projections, or (C and E and F) single plane images, representative of 101 dsRed-VPS4B E235Q and 153 EGFP-VPS4B E235Q CTL-target conjugates from three biologically independent experiments. Scale bar, 5 µm. (**G** and **H**) Quantitation of "immune synapses" (contacts) formed by CTLs expressing dnVPS4 showing (G) percentage CTLs able to recruit a signaling protein (Lck, PKCθ or Zap70) to the "immune synapse" at the contact site formed between targets and CTLs expressing dsRed-tagged dnVPS4 (n = 90) or EGFP-tagged dnVPS4 (n = 143) compared to CTLs expressing pmaxGFP (n = 194). (H) Percentage of conjugates with CD3ε-ectosomes transferred to targets where conjugated CTLs (+) expressed constructs (ds Red, n = 90; EGFP, n = 143) or (-) showed no expression (dsRed, n = 571; EGFP, n = 360), as shown. Graphs are representative of three independent experiments. Error bars show mean values ± SD with each data point representing cells from 2-10 fields across a single distinct sample, over two independent experiments.

Movie S1. Corresponds to fig. S2

FIB-SEM image sequence through a CTL expressing Lifeact-APEX (central cell) interacting with two targets. Lifeact-APEX is present across the entire late contact site with the target on the left dying (indicated by the vacuolated ER) while Lifeact-APEX is present in long projections with the target on the right (e.g. t = 00.00.42) but depleted from the small area of flattened membrane (see t = 00.00.40).

Movie S2. Corresponds to Fig. 2 A to C

Tomogram reconstructed from 4 sequential thick sections (~250 nm) through a ~1 μm depth of the immune synapse formed between a CTL expressing CD3ζ-APEX left/bottom cell) and target (right/top cell), with CD3ζ-APEX DAB reaction product traced in green and the CTL membrane in magenta. CD3ζ-APEX labels flattened CTL membrane at the contact site. CD3ζ-APEX is also seen in endocytic tubules internalising back into the CTL (e.g. t = 00.00.11 to 15, t = 00.00.25 to 29) and ectosomes at the periphery of the flattened labeled membrane (e.g. t = 00.00.08 to 11, and from 00.00.13).

Movie S3. Corresponds to Fig. 2 A to C

3D model generated from the tomogram and tracing shown in Movie S2 revealing, sequentially, total CTL membrane (magenta, t = 00.00.00), CTL membrane labeled with CD3ζ-APEX (green, t = 00.00.14), CD3ζ-APEX-labeled ectosomes (green, t = 00.00.29) and the target membrane (blue t = 00.00.43).

Movie S4. Corresponds to Fig. 3 A to D

Tomogram reconstruction of 5x ~250 nm sequential thick sections through a ~1.25 μm depth of the immune synapse formed between a CTL expressing CD3ζ-APEX (left cell) and target (right cell). CD3ζ-APEX DAB reaction product shows only residual labeling of the CTL membrane (at small CTL-target contact sites e.g., top, t = 00.00.06 to 17) and instead is accumulated in ectosomes in the extracellular space between the CTL and target, and internalised into targets (e.g., t = 00.00.10, 00.00.16, 00.00.19 to 20).

Movie S5. Corresponds to Fig. 3 A to D

3D model generated from the tomogram in Movie S4 showing sequentially the total CTL membrane (magenta, t=00.00.00), residual CTL membrane patches labeled with CD3ζ-APEX and budding CD3ζ-APEX-labeled ectosomes (green, t = 00.00.14), extracellular CD3ζ-APEX-labeled ectosomes (green, t = 00.00.29) and the target membrane (blue t = 00.00.43).

Movie S6. Corresponds to Fig. 3 E to F

Tomogram reconstructed from 4x ~250 nm sequential thick sections through a ~1 μm depth of the immune synapse formed between a CTL expressing CD3ζ-APEX (left cell) and target (right cell),

showing CD3ζ-APEX DAB reaction product concentrated in ectosomes in the extracellular space (cleft) opposite the polarised centrosome.

Movie S7. Corresponds to Fig. 3 E to F

3D model generated from the tomogram in Movie 4 showing sequentially the total CTL membrane (magenta, t = 00.00.00), the cSMAC labeled with CD3ζ-APEX (green, t = 00.00.14), extracellular CD3ζ-APEX-labeled ectosomes (green, t = 00.00.28) and the target membrane (blue t = 00.00.43).

Movie S8. Corresponds to fig. S9

Live confocal imaging of a CTL expressing PKCε-GFP loaded with cgSAG after uncaging with 405 nm laser exposure as described in methods and fig S9. Imaging started 10 s after uncaging. (25 x real time).

Movie S9. Corresponds to Fig. 5C

Live confocal imaging of a CTL expressing PKCε-GFP contacting an EL4 target cell (blue), shown as a projection of 33 z-planes, with images taken every 13.5 sec for 13 min. PKCε-GFP-labelled ectosomes form from the labeled synapse and are transferred to the target. (67 x real time).
